# Integration of anatomy and physiology into nursing practice as perceived by undergraduate students and registered nurses: a scoping review

**DOI:** 10.1186/s12912-023-01436-0

**Published:** 2023-08-17

**Authors:** Miwa Horiuchi-Hirose, Tomoko Fukuoka, Yuka Saeki

**Affiliations:** 1 Department of Health and Nutrition, Tokiwa University, Mito, 310-8585 Ibaraki Japan; 2 Former Department of Nursing, Ibaraki Christian University, Hitachi, 319-1295 Ibaraki Japan; 3 Graduate School of Medicine, Ehime University, Shitsukawa, Toon, 791-0295 Ehime Japan

**Keywords:** Anatomy and physiology, Nursing, Education

## Abstract

**Objective:**

The current study aimed to determine perceptions of registered and student nurses regarding the integration of anatomy and physiology into nursing practice.

**Methods:**

This scoping review was conducted following the checklist provided in the Reporting Items for Systematic reviews and Meta-Analysis extension for scoping reviews. Articles published in PubMed, ERIC, and CINAL from January 1, 2002 to September 30, 2022 were included.

**Results:**

A literature review of 20 articles that matched the indicative criteria revealed that both undergraduate student and registered nurses recognized knowledge of anatomy and physiology as important to nursing practice. Student nurses recognized that such knowledge is related to understanding patient pathophysiology, patient observation, treatment selection, and patient safety and forms the basis for nursing practice. Registered nurses who were confident in their knowledge of anatomy and physiology also reported that they were able to explain the rationale for their nursing practice. They also reported that this knowledge is necessary for communication with multiple professions, which promotes patient/family trust in nurses and is the basis for building trusting relationships with patients and their families.

**Conclusions:**

Although undergraduate student and registered nurses recognized the importance of learning anatomy and physiology, the integration of anatomy and physiology into nursing practice was not the same for all student and registered nurses. This suggests the need to investigate the overall perceptions of nurses regarding the integration of anatomy and physiology into nursing practice and for faculty to discuss how to facilitate critical thinking among students.

## Introduction

The exponential growth of information in health care knowledge and the complexity of medical sciences have required clinical nurses to be not only skillful but also knowledgeable on the scientific basis of diseases and treatment [1]. Knowledge regarding anatomy and physiology is essential for understanding human beings and is of great importance in nursing practice, especially in clinical decision making [2]. A qualitative research study by Van Wissen et al. [3], which sought to determine the impact of studying anatomy and physiology at a postgraduate level by disseminating pre- and post-course semi-structured questionnaire for an anatomy and physiology course as part of a Master’s Degree Program in Nursing, revealed improvement in confidence, particularly in communication, linking nursing theoretical knowledge to practice, and clinical nursing knowledge. However, with only a few published studies have been available regarding this field of research in undergraduate student nurses [4]. The overall view regarding how knowledge of anatomy and physiology relates to nursing practice remains unclear. This leads us to believe that faculty members teaching anatomy and physiology may not have a clearly defined perspective on student achievement goals for their anatomy and physiology courses. Student nurses seem to exhibit better academic performance level when they are well prepared and can understand the relevance of anatomy and physiology in nursing practice [4]. However, student nurses have perceived anatomy and physiology courses to be much more difficult than other subjects [5].

Studies have shown the effectiveness of blended learning, flipped learning, and other teaching methods in anatomy and physiology education. Blended learning has been reported to improve nursing students’ performance in anatomy and physiology, self-reported learning outcomes, and high levels of satisfaction [6]. The benefits of blended learning include flexible study time and improved independent study skills [7]. A study on flipped learning showed that the results for the flipped classroom group exceeded those for the traditional lecture group, with 70% of the student nurses reporting satisfaction with the flipped classroom method, indicating that it enhanced their learning and increased their interest in the course [8]. However, the primary focus of these teaching methods is not so much on promoting thinking that relates anatomy and physiology knowledge to nursing practice, but rather on how to consolidate anatomy and physiology knowledge.

With the current approach toward anatomy and physiology education, there is a need to examine educational methods that integrate anatomy and physiology knowledge into nursing practice and evaluate the effectiveness of such methods. As such, it is necessary to (1) investigate how students and nurses perceive anatomy and physiology learning and the integration of anatomy and physiology into nursing practice and (2) define the achievement goals of anatomy and physiology education required by students enrolled in a bachelor’s program.

The nursing education system in the United Kingdom, Australia, and New Zealand, among other countries, have included anatomy and physiology under bioscience, an umbrella term that includes anatomy, genetics, microbiology, physiology, pharmacology, and pathophysiology [9]. However, given that other countries use the term anatomy and physiology, this term was used in the current study.

### Scoping review objective

This scoping review aimed to summarize the perceptions of undergraduate student and registered nurses on anatomy and physiology learning and the integration of anatomy and physiology learning and nursing practice.

### Scoping review question

What are the perceptions of student and registered nurses on learning anatomy and physiology? What are the perceptions of undergraduate student and registered nurses on the integration of anatomy and physiology learning and nursing practice in undergraduate nursing education?

## Methods

### Protocol and registration

The review protocol for this study was not published. This scoping review was conducted following the Preferred Reporting Items for Systematic reviews and Meta-Analysis extension for scoping reviews PRISMA-ScR Checklist [10] and follows the five-strategy review process as identified [11] and later refined by Lavec [12] and Peters [13].

### Eligibility criteria

Titles/abstracts and full texts were screened to optimize literature coverage using the eligibility criteria shown in Table 1.

**Table 1:** Inclusion and exclusion criteria

	Inclusion	Exclusion
Language	English	Non-English
Time Period	Studies published from January 1, 2002 to September 31, 2022	Any study published before 2001
Study Focus	Students’ and nurses’ perceptions regarding anatomy and physiology learning Students’ and nurses’ perceptions about integrating anatomy and physiology learning into nursing practice	Any study not mentioning students’ and nurses’ perceptions regarding anatomy and physiology learning Any study not mentioning students’ and nurses’ perceptions about integrating anatomy and physiology learning into nursing practice
Study Design	Any	None

Reviews, brief reports, conference notes/abstracts, and comment articles were excluded. Studies that focused on the perceptions of nurses and student nurses regarding anatomy and physiology learning and those on the perceptions of nurses and student nurses about integrating anatomy and physiology learning into nursing practice were considered eligible. Papers that did not fit these criteria were excluded.

### Information sources

An electronic search of PubMed, ERIC, and CINAL was conducted in October 2022.

### Search

The search terms used were “nurse,” “nursing,” “anatomy,” “physiology,” “bioscience,” and “student.” Using Boolean combinations of the primitives, our final string was (nurse or nursing) AND (anatomy or physiology or bioscience) AND student.

Because nursing education curricula have changed with changing social conditions, in order to analyze the most recent research findings, the period of publication was limited to the last 10 years, and articles were limited to those published between January 1, 2002 and September 31, 2022. Although it is strongly recommended that scoping reviews should not be limited by language selection criteria [11], they were included in the exclusion criteria for this study due to the extreme difficulty of translating and understanding non-English speaking nurse education curricula. The seven excluded articles were in Chinese, Portuguese, and Israeli.

### Data charting process and items

Microsoft Excel spreadsheets were used by the lead author to chart the characteristics of all articles. Table 2 was developed to chart the following key information from the selected articles: author(s) and year of publication, sample, objective, methodology, summary of findings, and country. Data from each included article were extracted by an independent researcher and counter-evaluated for accuracy or missing information by another researcher. Selected data with counter-information were re-evaluated by the entire team, after which a consensus was reached by the team to regarding the final charted data.

**Table 2:** Student’ and Nurses’ perceptions of anatomy and physiology (A&P) learning and integrating A&P learning into nursing practice

Author(s)	Year	Sample	Objective	Study design	Summary of findings	Country	Content focus
Andrew, McVicar, Zanganeh and Henderson	2015	97 (first semester of the first year) 82 (first semester of the second year)	To investigate students’ self-efficacy for science and their perceptions of learning A&P.	A prospective correlational survey	Students with high self-efficacy valued science and were expected to succeed in their A&P courses more than those with low self-efficacy.	New Zealand	Student’ perceptions of A&P learning
Barton, Bentley, Craft, Dupen, Gordon, Cayanan, Kunst, Connors, Todorovic and Johnston	2021	30 undergraduates and postgraduate nursing students (focus group interview) 406 undergraduates and postgraduate nursing students (questionnaires)	To investigate the student’s perception of the relationship between clinical relevance and engagement with A&P content (attention and time).	Cross-sectional survey	The clinical relevance of A&P was widely appreciated, with 91.6% of the undergraduate nursing students indicating that every nurse must have a good understanding of A&P. A&P content was time-consuming for 50% of the undergraduate nursing students.	Australia	Student’ perceptions of integrating A&P learning into nursing practice
Behrendt, Foster and Machtmes	2020	283	To identify students’ perceptions of what is required for success in anatomy and physiology courses.	Descriptive qualitative design (Case study design)	In order to pass the anatomy and physiology courses, students must attend every class, actively participate, take advantage of office hours, and understand the subject rather than simply memorizing it.	US	Student’ perceptions of A&P learning
Birks, Ralph, Cant, Chun Tie and Hillman	2018	1583	To determine the existence and scope of the theory- practice gap in relation to A&P in nursing.	Cross-sectional survey	During their school years, registered nurses place a high value on expanding their A&P knowledge. Anatomy, physiology, and pathophysiology courses were particularly given importance.	Australia	Nurses’ perceptions of A&P learning
Christensen, Craft, Wirihana and Gordon	2015	43	To explicitly demonstrate the connections between physiology, pathophysiology, and nursing practice.	Descriptive qualitative design	More than 90% of the respondents agreed that having a bioscientist who focussed on the needs of the nursing students helped to increase the knowledge of patient conditions. The teaching provided by the bioscientist was beneficial in relating physiology and pathophysiology to nursing care.	Australia	Student’ perceptions of integrating A&P learning into nursing practice
Choi-Kwon, Song, An and Choe	2002	559	To clarify the importance of A&P from the nurses’ perspective.	Descriptive qualitative design	Many registered nurses consider their A&P knowledge inadequate while implementing the nursing process, communicating with other medical personnel, or teaching patients.	Korea	Nurses’ perceptions of integrating A&P learning into nursing practice
Craft, Hudson, Plenderleith, Wirihana and Gordon	2013	273	To investigate incoming students’ perceptions, knowledge, and approaches to learning A&P.	Descriptive qualitative design	Many students have reservations about studying A&P and consider it is more difficult and demanding than nursing courses. They also feel that there is more content to learn in a A&P course than in a nursing course.	Australia	Student’ perceptions of A&P learning
Craft, Hudson, Plenderleith and Gordon	2017	22	To investigate the reflections of new graduate registered nurses’ on A&P courses taken during their nursing program as well as the relationship between A&P content and clinical practice.	Descriptive, cross-sectional survey	Registered nurses believe that they lack the confidence to explain their knowledge of A&P. They are been to improve their knowledge and agree that A&P coursework should be extended up to the final year of undergraduate study. Moreover, they believe that it is important to relate A&P knowledge to nursing practice.	Australia	Nurses’ perceptions of A&P learning Nurses’ perceptions of integrating A&P learning into nursing practice
Davis	2010	42	To determine whether the nurses considered A&P studies in their preregistration nursing courses to be sufficient, associated with enough practical exposure, and prepared them for their roles as registered nurses.	Case study methodology	51% of the registered nurses indicated that the content of A&P during their student years was limited. In addition, 40.5% of the registered nurses believed that the A&P content taught during their school was incompatible with nursing practice.	UK	Nurses’ perceptions of integrating A&P learning into nursing practice
Evensen, Brataas and Cui	2020	57 (first year)	To investigate their motivations, academic performances, and reactions to different teaching methods in an anatomy and physiology course.	Descriptive small-scale study	The nervous system, kidneys and the urinary tract, and acid-base balance were the most difficult topics. Independent study was significantly associated with higher A&P examination grades.	Norway	Student’ perceptions of A&P learning
Fell, Dobbins and Dee	2016	17 final year students (focus group interview) 112 final year students (questionnaires)	To explore the experiences of A&P learning in clinical placement.	Descriptive qualitative design	97% of the students clearly appreciated the importance of A&P knowledge in nursing practice. Students frequently emphasized the association between fundamental A&P knowledge and understanding conditions, observations, and treatment options. Students emphasized the importance of A&P knowledge in supporting patient care, as well as the link between A&P knowledge and patient safety.	UK	Students’ perceptions of integration of A&P learning and nursing practice
Friedel and Treagust	2005	155 students 29 nurse educators	To investigate the framework for A&P in nursing education programmes in New Zealand, and the perceptions that nurse educators, nursing students and nursing graduates have in relation to A&P in the nursing curriculum.	Interpretative survey strategy	Although many students perceived A&P to be difficult and anxiety-provoking, they also perceive it to be a very important and valuable part of their nursing program.	New Zealand	Student’ perceptions of A&P learning
Gordon, Hudson, Plenderleith, Fisher and Craft	2017	126 final year students	To examine students’ experiences with A&P, with a particular emphasis on the relationship to nursing practice.	Cross-sectional survey	The majority of participants considered A&P subjects required more work than nursing subjects (65.9%), and they would like a better understanding of A&P (73.8%); however, they also understood that A&P is the foundation of nursing practice (76.2%). Participants without any secondary school science education were unable to apply A&P concepts to patient conditions.	Australia	Student’ perceptions of A&P learning Students’ perceptions of integration of A&P learning and nursing practice
Logan and Angel	2011	85 questionnaires 15 focus group interviews	To investigate the science–nursing tension and impact on nursing students studying A&P.	Descriptive qualitative design	Practicum supervisors who have their own knowledge of A&P provide opportunities for students to integrate that knowledge into nursing practice.	Australia	Nurses’ perceptions of integrating A&P learning into nursing practice
Mckee	2002	104–211 first year students	To determine whether the admission criteria and study habits have a significant influence on A&P examination results.	Descriptive qualitative design	Pre-enrollment biology experience, biology achievement, class attendance, and the use of core texts had a significant influence on A&P examination results.	Ireland	Student perceptions of A&P learning
McVicar, Clancy and Mayes	2010	19 interviews with nurses (stage1) 10 questionnaires with lectures (stage2)	To investigate how nurses’ knowledge of the A&Ps is embedded in their practice and the manner of communication.	Descriptive qualitative design	Many registered nurses consider their A&P knowledge to be inadequate, although they believe that nursing practice is the most important aspect of A&P learning.	UK	Nurses’ perceptions of integrating A&P learning into nursing practice
Molesworth and Lewitt	2016	7 first year students	To investigate students’ experiences with A&P learning, teaching, and application in the context of practice setting.	Descriptive qualitative design (Phenomenological study)	Although participants recognized the importance of A&P in clinical settings, they found that learning opportunities were often limited. To enhance A&P learning, participants expressed a desire for a more structured and an integrated approach, both in practice and in university settings along with additional peer learning opportunities.	Scotland	Students’ perceptions of integration of A&P learning and nursing practice
Montayre, Dimalapang, Sparks and Nevill	2019	540	To investigate students’ perceptions of A&Ps in New Zealand undergraduate nursing programs in terms of relevance to practice, teaching delivery, self-competence, and challenges encountered.	Descriptive, cross-sectional survey design	The perceived importance of A&P and the difficulties encountered in learning differ between younger and older students. Positive perceptions of A&P become apparent when nursing students reach their final year of study.	New Zealand	Student perceptions of A&P learning
Montayre, Ramjan, Maceri and Salamonson	2021	15	To investigate new registered nurses’ experiences with applying A&P concepts in their day-to-day nursing practice.	Descriptive qualitative design	A&P knowledge gives a preceptor confidence. Registered nurses who are self-assured in their A&P knowledge can explain the evidence for their nursing practice. They stated that it helped them gain confidence as registered nurses and served as the foundation for developing trusting relationships with patients and their families.	Australia	Nurses’ perceptions of integrating A&P learning into nursing practice
Mortimer-Jones, Wall and Russell	2018	188 first year students	To determine whether there was a difference in nursing students’ anxiety levels during the A&P laboratory classes versus the clinical laboratory classes.	Descriptive qualitative design	There were no statistically significant differences in anxiety levels between the A&P and clinical laboratory classes.	Australia	Student perceptions of A&P learning

Anatomy and physiology; A&P

### Data synthesis

A scoping review was employed to identify all relevant literature, followed by a narrative synthesis. Due to heterogeneity across studies and even within studies with similar methodologies, metasynthesis for qualitative data was not possible. Instead, studies were combined to summarize the study characteristics, followed by a textual narrative synthesis. This approach helped arrange disparate study types into more homogenous subgroups, thereby aiding in the synthesis of different types of evidence. Study characteristics, context, quality, and findings have been reported according to a standard format, and their similarities and differences have been compared across studies [14].

## Results

### Search outcomes

**Fig. 1:**
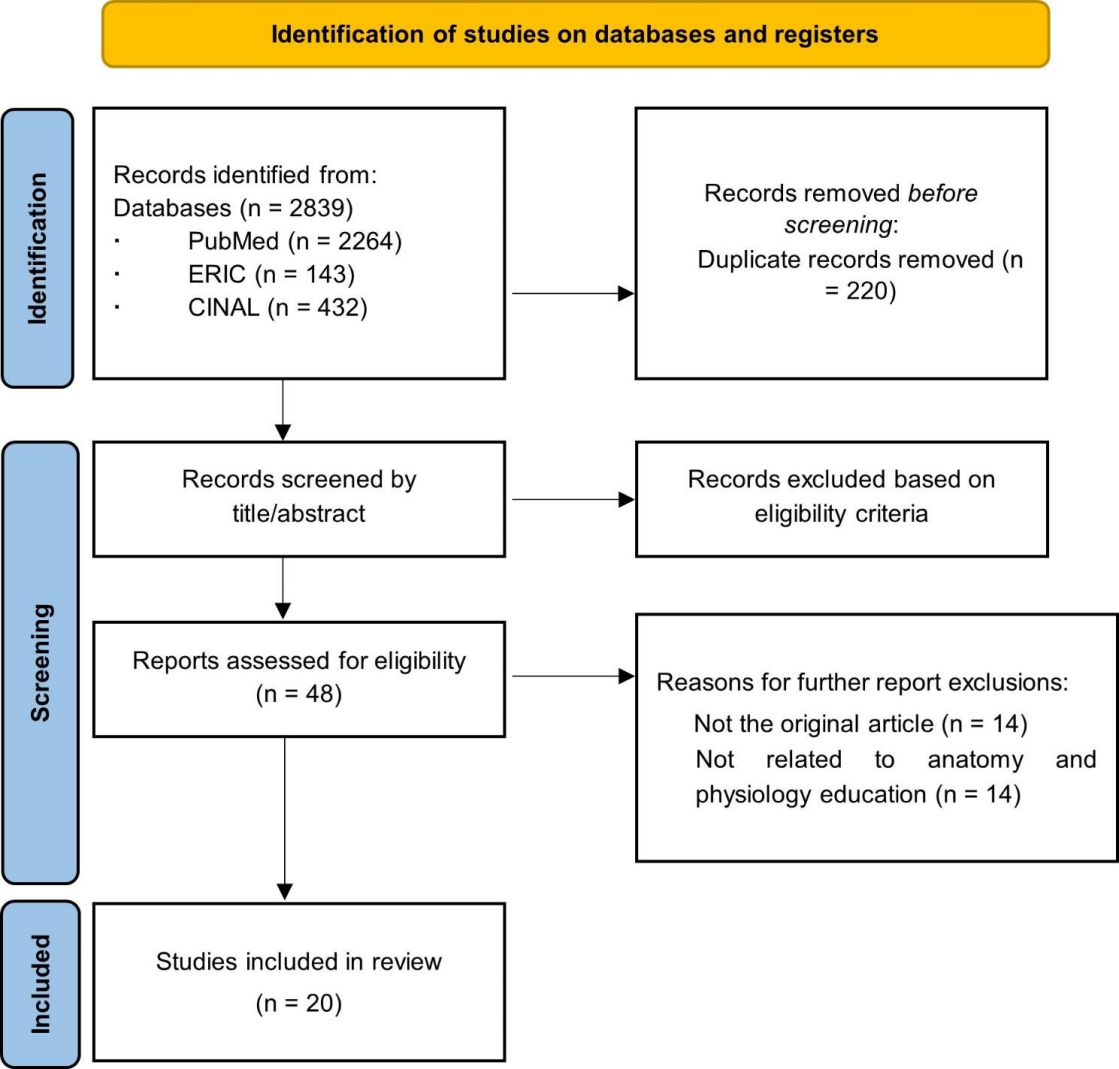
PRISMA flow diagram for the study search and selection process (diagram based on that of Page et al. [15])

Search results were exported to the bibliographic software program EndNote (Clarivate Analytics, Philadelphia, PA, USA). Duplicate articles were removed using the literature management software. Data were extracted from the literature management software to Microsoft Excel. Then, two researchers (MH and TF) independently extracted data from the titles and abstracts based on our eligibility criteria. Any disagreements were resolved through discussion between the two authors. Figure 1 presents the PRISMA flowchart [15] for the study selection process. A total of 2,839 papers were obtained, from which 220 duplicates were eliminated. The number of papers subjected to the title and abstract review was 2619, and the number of papers excluded from title and abstract review was 2571. Further screening of the full text of the remaining 48 articles resulted in the exclusion of 28 articles that were either not original papers or were unrelated to anatomy and physiology education. The excluded papers included 14 nonoriginal reports, 13 cadaveric dissections, and 1 patient simulation. Finally, 20 articles met the inclusion criteria (Fig. 1).

A total of nine studies were from Australia; three from the UK; three from New Zealand; and one each from Norway, Korea, Ireland, Scotland, and the USA. Among the 20 articles reviewed, 3 were qualitative, 11 were quantitative, and 6 were mixed methods studies. The study participants were registered nurses in 7 papers and undergraduate student nurses in 13 papers.

### Study of anatomy and physiology

Impressions of undergraduate student nurses on anatomy and physiology were as follows: there was much content to learn [16], it was difficult [16, 17], and it required a considerable amount of study time [9, 18]. In addition, Montayre [19] reported that the perceived difficulty of anatomy and physiology was replaced by positive perceptions in the final year. The impressions of registered nurses on anatomy and physiology were as follows: courses in anatomy, physiology, and pathophysiology were rated as particularly high priority for learning in the education of student nurses [1]. However, 40.5% of registered nurses perceived that their anatomy and physiology classes as students were not compatible with actual nursing practice [20].

Effective learning as perceived by undergraduate student nurses was reported as independent study [17], class attendance [21, 22], active participation in class [21], use of office hours [21], and use of core textbooks [22]. One study reported that understanding the content was important, not memorizing it [21]. Collaborative classes between anatomy and physiology faculty and nursing specialty area faculty [23], as well as collaborative learning opportunities between field placement sites and the university [24], were evaluated highly by the students. Registered nurses agreed that the study of anatomy and physiology should be extended to the final year of undergraduate study [25].

Undergraduate student nurses with high self-efficacy were more science-oriented and had higher expectations for success in the bioscience course than were students with low self-efficacy [26]. Several students had anxiety about studying bioscience [16, 27]. However, Mortimer-Jones [28] reported no significant difference in anxiety scores between bioscience and clinical classes.

Several registered nurses perceived their knowledge as low [29] and considered their anatomy and physiology knowledge inadequate for conducting the nursing process, communicating with other health care professionals, and teaching patients [30]. Registered nurses also indicated that they lacked confidence in explaining their knowledge of anatomy and physiology as evidence of nursing practice [25] and wanted to develop more knowledge of these topics [9, 25].

### Integration of anatomy and physiology into nursing practice

Undergraduate student nurses [18, 31] and registered nurses [22, 25] recognized that knowledge of anatomy and physiology was important to nursing practice. The students recognized that such knowledge was important for understanding patient pathophysiology, patient observation, treatment selection, and patient safety [31] and that it forms the basis for nursing practice [9]. Registered nurses who were confident in their anatomy and physiology knowledge were also capable of explaining the rationale for their nursing practice. They also reported that this promotes patients/family trust in nurses, which they perceived as the basis for building trust with patients and their families [31]. In addition, registered nurses’ knowledge of anatomy and physiology gave them the confidence to serve as preceptors who influence students’ practice [32], and practice instructors with sufficient knowledge were able to provide students with opportunities to integrate that knowledge with their nursing practice [33].

## Discussion

### Summary of evidence

This review summarized the perceptions of undergraduate student and registered nurses on anatomy and physiology education in undergraduate nursing programs, as well as their perceptions on the integration of anatomy and physiology into nursing practice. The diversity of assessment methods among the 20 studies, including qualitative, quantitative, and mixed methods, may have affected the aggregated data.

### Establishment of anatomy and physiology knowledge

Although undergraduate student nurses initially perceived anatomy and physiology as difficult [16, 17], this perception changed for the better in their final year [19] given that clinical practice allowed students to use their knowledge of anatomy and physiology to sharpen their critical thinking skills and clinical judgments [19].

Encouraging class participation [21, 22], use of office hours [17], and independent study is important so that students’ learning is not hindered by their impression that learning anatomy and physiology is difficult and their learning continues throughout their final year of study [25]. With regard to continuity of learning, Spencer [34] believes that continuity of learning, for example, in the training of physicians, will deepen students’ understanding of clinical medicine and how anatomy and physiology and specialized subjects are integrated. Additionally, reports have shown the effectiveness of collaborative teaching between faculty teaching anatomy and physiology and those faculty teaching specialized nursing courses [23]. Anatomy and physiology are often taught by a diverse group of faculty members, including clinicians, instructors from non-nursing colleges, and instructors with national nursing certifications, several of whom have no experience in clinical nursing practice [35]. As such, some classes are taught without an understanding of how anatomy and physiology relate to nursing practice. However, studies have shown that when anatomy and physiology is taught by nationally certified nursing faculty, students’ knowledge of anatomy and physiology was insufficient, and that such students would likely not understand basic anatomy and physiology knowledge of diseases and disorders when taking clinically oriented nursing courses [36]. Given that anatomy and physiology courses importantly serve as the foundation for nursing practice, collaboration between faculty members in charge of anatomy and physiology courses and those in charge of specialized subjects should be required based on the curriculum of each university. In addition, it is important for faculty members in charge of teaching anatomy and physiology to exchange information with faculty members of other universities in order to share effective methods for teaching the course.

### Collaboration with clinical nursing instructors

Integration of anatomy and physiology into nursing practice is best achieved through clinical practice experiences. Furthermore, studies have reported that students’ thought processes are enhanced in clinical practice settings wherein the clinical instructor has sufficient knowledge of anatomy and physiology [24]. For students to learn effectively, the cooperation of not only the faculty but also the practicum supervisor is essential [24]. Evidence suggests that clinical nurses also want to further develop their knowledge of anatomy and physiology [9, 25]. Nurses who are confident in their knowledge of anatomy and physiology have a positive influence on the clinical practice of students [32]. We believe that providing clinical nurses with opportunities to study anatomy and physiology in the future will facilitate the continuous improvement of student nurses’ practical skills. Future studies need to verify the effectiveness of clinical practice initiatives and new education systems on campus to facilitate the integration of anatomy and physiology knowledge into nursing practice. Research physicians who specialize in basic science or clinically oriented basic science play an important role in bridging the gap between basic specialty and clinical disciplines [34]. Perhaps inviting more such educators to participate in nursing student education could help integrate anatomy and physiology into nursing practice.

### Integration of anatomy and physiology and nursing practice

Apart from acquiring systemic knowledge, it is necessary to visualize how this knowledge is connected to nursing practice and evaluate students’ academic achievement.

Studies have suggested that knowledge of anatomy and physiology is associated with patient safety [31], decision making, and building trust [32]. However, given the limited research reports on these perspectives, there is a need to understand the overall picture in the future and visualize how knowledge of anatomy and physiology is specifically linked to nursing practice. To link knowledge of anatomy and physiology to nursing practice, it is important to clarify the goals within each subject while considering the curriculum of each university, so that students themselves are aware of what they should learn. The use of clinical material created for medical students’ anatomy and physiology courses increases the relevance of the course to students and improves students’ knowledge retention [34].

In the future, research will need to facilitate thinking that enhances the effectiveness of learning and integrates it with nursing practice, examine educational content and methods, and evaluate their effectiveness. Therefore, appropriate evaluation of the achievement objectives for anatomy and physiology is necessary to clarify how learning anatomy and physiology facilitates nursing practice and determine how to promote sufficient learning among students.

### Limitations

For this scoping review, database searches were limited to studies pertaining to education in anatomy, physiology, and biological sciences, and keywords such as “pathophysiology” and “microbiology” were not used. National nursing school designations vary, and curricula are uniquely developed by each training school. Because the design of anatomy and physiology courses (i.e., textbooks, time, teaching methods, and examination methods) varied among schools, a critical evaluation was not conducted. Because this aimed to identify perceptions regarding the integration of anatomy and physiology learning into nursing practice, the quality of each literature review was not evaluated. The student and nurse perceptions in this study may have been influenced by differences in the course design.

## Conclusions

Regarding perceptions on integrating anatomy and physiology into nursing practice, students reported that anatomy and physiology knowledge was important for patient safety and understanding the patient’s condition, whereas nurses reported that such knowledge was related to explaining the rationale of nursing practice, building trust, and effective practice teaching. In the future, research needs to clarify the attainment goals related to the integration of anatomy and physiology into nursing practice for students enrolled in a bachelor’s program. It is also necessary to establish an anatomy and physiology education system that connects anatomy and physiology with nursing practice and to examine the effectiveness of such an education system.

## Data Availability

All data generated or analyzed during this study are included in this published article.
